# A Novel Case of Acquired Isolated Left Ventricular Non-compaction in a Primigravida: Revisiting the Diagnostic Criteria of Left Ventricular Non-compaction

**DOI:** 10.7759/cureus.33823

**Published:** 2023-01-16

**Authors:** Esiemoghie J Akhigbe, Ebubechukwu Ezeh, Nadew Sebro, Olalekan Olanipekun, Carlos Rueda Rios

**Affiliations:** 1 Internal Medicine, Marshall University Joan C. Edwards School of Medicine, Huntington, USA; 2 Cardiovascular Medicine, Marshall University Joan C. Edwards School of Medicine, Huntington, USA

**Keywords:** heart disease in pregnancy, acquired heart disease, genetics, cardiomyopathy, left ventricular non-compaction, heart failure

## Abstract

Left ventricular non-compaction (LVNC) is rare cardiomyopathy characterized by the presence of a two-layered myocardium with prominent trabeculations. It has high rates of mortality and morbidity. Clinical presentation could vary from asymptomatic patients to developing ventricular arrhythmias, thromboembolism, heart failure, and even sudden cardiac death.

We present a 23-year-old primigravida with a childhood history of dilated cardiomyopathy secondary to post-viral myocarditis presenting at 32 weeks gestation with dyspnea on exertion. Initial 2-D echocardiogram revealed a mildly dilated left ventricle with apical trabeculation and a 2-layer distinction between compacted and noncompacted myocardium indicating non-compaction of the left ventricle.

This case presents a peculiar confluence of cardiac genetics, normal physiology, and infection. We describe a rare form of acquired LVNC that transformed from another type of cardiomyopathy to LVNC during pregnancy drawing attention to the causality pathways of LVNC.

## Introduction

Left ventricular non-compaction (LVNC) is a rare congenital cardiomyopathy identified by the presence of very prominent recesses in the left ventricle. These intertrabecular recesses are known to have bidirectional flow within the ventricular cavity rather than in the coronary circulation [1]. It is presumed LVNC is caused by the arrest of the normal development of the heart during embryogenesis. Multiple studies have highlighted the importance of genetics in the development of this pathology. During the management of individuals with confirmed cases of LVNC genetic studies are recommended in first-degree relatives to help with early diagnosis of potential risk and gene localization [2]. The diagnosis of this disease is made primarily by imaging with the most commonly used modalities being echocardiography, contrast ventriculography, computed tomography (CT), and magnetic resonance imaging (MRI) [3].

Despite the advances in technology, there has been limited data on the incidence and pathogenesis of acquired LVNC and thereby hindering adequate diagnosis and management. This case illustrates an unusual presentation of acquired LVNC in a young primigravida hereby raising concern for the importance of updated management and diagnostic guidelines.

## Case presentation

A 23-year-old primigravida with a past medical history of dilated cardiomyopathy secondary to post-viral myopathy after a childhood infection with a respiratory syncytial virus (RSV). Home medications include furosemide 20 mg daily. She presented to the emergency department (ED) at 32 weeks completed gestation with new complaints of worsening shortness of breath, intermittent chest pain, and dyspnea on exertion. Her symptoms progressively worsened to the point where is only able to walk a flight of stairs before becoming short of breath (NYHA3b). The patient also had an associated history of 2-pillow orthopnea and bilateral pedal edema which worsened as the day progresses and reduces upon elevation. She also endorses episodes of cough which is nonproductive in nature. She denied any paroxysmal nocturnal dyspnea, palpitations, or syncope.

**Figure 1:**
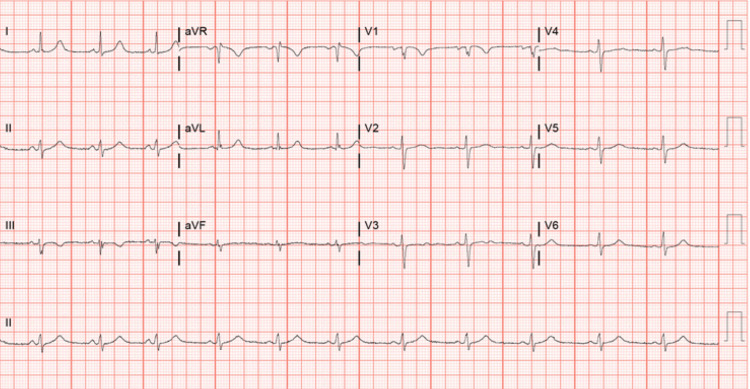
Electrocardiogram performed on admission showing normal sinus rhythm with no ST-T wave abnormalities.

**Figure 2:**
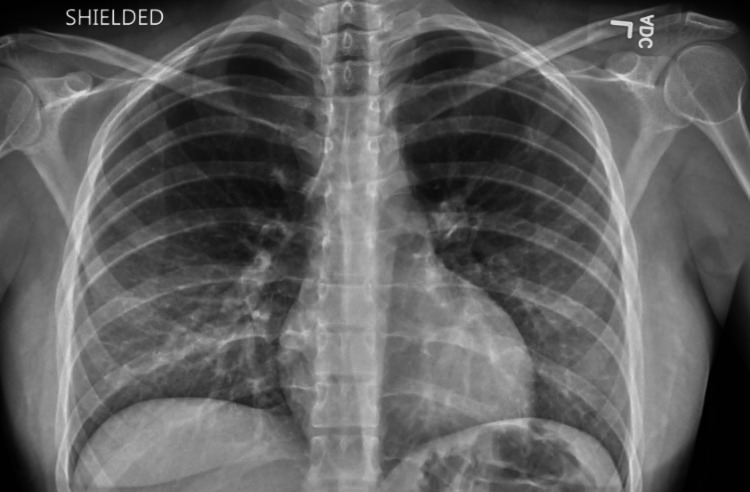
Antero-posterior chest radiograph showing minimal bilateral pleural effusion and pulmonary edema.

**Figure 3:**
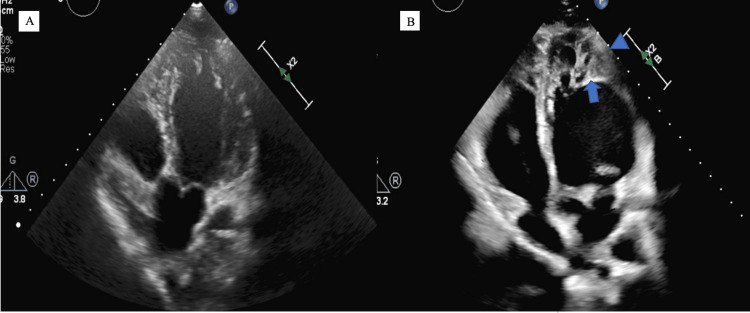
Echo scans. A) Apical 5-chamber view showing minimal trabeculations in the left ventricular apex. B) Repeat Echo showing increased trabeculation in the apex with a blue arrowhead showing area of compaction and full arrow showing area of non-compaction.

Upon arrival to the ED, the patient was hemodynamically stable with a blood pressure of 111/68 mmHg and a pulse rate of 73 bpm. The patient had SPo2 of 98% in ambient air. Initial laboratory investigation showed complete blood count (CBC), basic metabolic panel (BMP), and pro-B-type natriuretic peptide (Pro-BNP) were within normal limits. The electrocardiogram showed a normal sinus rhythm with no abnormalities (Figure 1). Chest X-ray showed a small bilateral pleural effusion with mild pulmonary edema (Figure 2). A 2-D echocardiogram performed at this encounter revealed a mildly reduced left ventricular ejection function (LVEF) of 45% and a mildly dilated left ventricle with apical trabeculations and non-compaction of the left ventricle (Figure 3B). In comparison, an Echo done approximately 6 months ago showed an LVEF of 49% with no obvious structural abnormality (Figure 3A). Also, a cardiac MRI done 2 years prior showed no evidence of any myocardial abnormality.

A diagnosis of pregnancy-induced LVNC was made and the patient was started on IV furosemide and was diuresised until she was euvolemic. Diuretics were then transitioned to their oral form and she was discharged with an increased dose of furosemide and a close follow-up in the heart failure clinic for further evaluation and genetic testing.

## Discussion

Isolated left ventricular non-compaction (ILVNC is known as a rare cardiomyopathy characterized by the presence of two layers of the myocardium with prominent recesses [1]. Although the exact cause is unknown, it is believed to be caused by the restriction of normal growth during embryogenesis of the endocardium and myocardium [2]. LVNC is usually classified as primary genetic cardiomyopathy [4]. The prevalence of LVNC was estimated to be <0.3% [1,3,5]. However, in more recent literature there has been a surge in the diagnosis of LVNC in patients with heart failure. Of these patients, about 35% of them have a high proportion of left ventricular trabeculations, and about 25% of these patients meeting the diagnostic criteria for LVNC irrespective of the criteria used [6]. Acquired LVNC has been recently reported to be associated with multiple autoimmune diseases like mitochondriopathy, myotonic dystrophy type essential thrombocythemia, and various neuromuscular disorders [1,5,7-9]. Although symptoms might range from benign to life-threatening, there has been an associated increase in the rates of mortality and morbidity in these patients. Other documented complications include life-threatening arrhythmias requiring implantable cardiac defibrillator (ICDs) placement, embolic events leading to strokes, and refractory heart failure requiring a cardiac transplant [10]. Despite all these advancements in understanding this enigmatic disease, there have been very few cases that have described LVNC in pregnancy thus making this particular case one of clinical and educational importance.

Echocardiography is considered the first line of diagnosis, however, a cardiac MRI is considered the gold standard in confirming the diagnosis of LVNC. These imaging modalities rely on the presence of LV myocardial trabeculations and the bi-layer between the compacted and noncompacted sections of the myocardium [9]. There are three different diagnostic criteria that have to date been published including Jenni et al., Chin et al., and Stollberger et al. [1,9,11,12]. Interestingly, our patient fulfills the Jenni et al. criteria for LVNC. The Jenni Criteria include (1) a two-layer structure, with a thin compacted layer and a thick noncompacted layer measured in end systole at the parasternal short-axis views; (2) absence of co-existing cardiac structural abnormalities; (3) numerous, excessively prominent trabeculations, and deep intra-trabecular recesses; (4) recesses supplied by intraventricular blood on color Doppler. The above criterion helps differentiate LVNC from postpartum cardiomyopathy (PPCM) where patients should only have heart failure in the last month of pregnancy or within 5 months of delivery and have no identifiable cause of heart failure or left ventricular dysfunction before the last 4 weeks of pregnancy. Our patient had a known history of cardiomyopathy, and initial cardiac imaging showed no identifiable LVNC. This is depicted by an echocardiogram performed 6 months prior to presentation with no overt signs of non-compaction thereby raising concerns for the acquired nature of this disease in this case.

Multiple studies have shown a risk of LVNC in physiological adaptation like in exercise. Of note, a study by Gati et al. involving over 1000 athletes demonstrated that about 18% of them exhibited an increase in LV trabeculations and a total of 8% fulfilled echocardiographic criteria for LVNC [13]. Although LVNC has continued to gain widespread recognition, however, its association with pregnancy has been limited. In pregnancy, it is known that there is an exponential increase in cardiac preload leading to increased left ventricular trabeculations [13]. This may lead to the conclusion that the increase in trabeculation is just a physiological response to the increased cardiac preload.

But then, in a prospective longitudinal echocardiographic study performed by Gati et al. which involved a total of 102 primigravida pregnant women (66 white, 36 black). The study showed that 25% of pregnant women developed increased left ventricular trabeculations, which was more common in African American women than in Caucasian women (47.2% versus 13.6%; P=0.0003). Of the total pregnancy cohort, 10 women (9.8%) fulfilled the Jenni et al. criteria, 19 (18.6%) fulfilled the Chin et al. criteria, and eight (7.8%) fulfilled both criteria for LVNC [14]. This supports the hypothesis that although the risk is minimal there is a risk of pregnancy triggering the onset of LVNC.

There are no clear specific guidelines for the management of patients with LVNC. However, treatment usually involves guideline-directed management for heart failure. The use of ICDs has been shown to prevent life-threatening arrhythmias and reduce mortality. There is also some debate on the use of anticoagulation to prevent embolic events. Anticoagulation is recommended in patients with a left ejection fraction of less than <40% [12,15].

## Conclusions

A delay in diagnosing patients with LVNC has been associated with worsening left ventricular systolic dysfunction, arrhythmias, and cardioembolic events resulting from thrombi. Although most women return back to baseline after 3-6 months following parturition, a few progress to worsening or persistent heart failure. This makes diagnosing and identifying women who meet these criteria for LVNC important, as it would aid in stratifying patients who would need long-term management.
